# Patterns of Fitbit Use and Activity Levels Throughout a Physical Activity Intervention: Exploratory Analysis from a Randomized Controlled Trial

**DOI:** 10.2196/mhealth.8503

**Published:** 2018-02-05

**Authors:** Sheri J Hartman, Sandahl H Nelson, Lauren S Weiner

**Affiliations:** ^1^ Department of Family Medicine and Public Health University of California San Diego La Jolla, CA United States; ^2^ University of California San Diego Moores Cancer Center University of California San Diego La Jolla, CA United States

**Keywords:** physical activity, technology, activity tracker, self-monitoring, adherence

## Abstract

**Background:**

There has been a rapid increase in the use of technology-based activity trackers to promote behavior change. However, little is known about how individuals use these trackers on a day-to-day basis or how tracker use relates to increasing physical activity.

**Objective:**

The aims were to use minute level data collected from a Fitbit tracker throughout a physical activity intervention to examine patterns of Fitbit use and activity and their relationships with success in the intervention based on ActiGraph-measured moderate to vigorous physical activity (MVPA).

**Methods:**

Participants included 42 female breast cancer survivors randomized to the physical activity intervention arm of a 12-week randomized controlled trial. The Fitbit One was worn daily throughout the 12-week intervention. ActiGraph GT3X+ accelerometer was worn for 7 days at baseline (prerandomization) and end of intervention (week 12). Self-reported frequency of looking at activity data on the Fitbit tracker and app or website was collected at week 12.

**Results:**

Adherence to wearing the Fitbit was high and stable, with a mean of 88.13% of valid days over 12 weeks (SD 14.49%). Greater adherence to wearing the Fitbit was associated with greater increases in ActiGraph-measured MVPA (b_interaction_=0.35, *P*<.001). Participants averaged 182.6 minutes/week (SD 143.9) of MVPA on the Fitbit, with significant variation in MVPA over the 12 weeks (F=1.91, *P*=.04). The majority (68%, 27/40) of participants reported looking at their tracker or looking at the Fitbit app or website once a day or more. Changes in Actigraph-measured MVPA were associated with frequency of looking at one’s data on the tracker (b=−1.36, *P*=.07) but not significantly associated with frequency of looking at one’s data on the app or website (*P*=.36).

**Conclusions:**

This is one of the first studies to explore the relationship between use of a commercially available activity tracker and success in a physical activity intervention. A deeper understanding of how individuals engage with technology-based trackers may enable us to more effectively use these types of trackers to promote behavior change.

**Trial Registration:**

ClinicalTrials.gov NCT02332876; https://clinicaltrials.gov/ct2/show/NCT02332876?term=NCT02332876 &rank=1 (Archived by WebCite at http://www.webcitation.org/6wplEeg8i).

## Introduction

The ubiquity of technology in day-to-day life is paving the way for new and emerging tools that can easily monitor physical activity. Use of commercially available, technology-based wearable activity trackers such as a Fitbit or Garmin is growing. A 2016 analysis revealed that 45% of American adults own at least one activity tracker, up from 21% in 2014 [1]. Trackers have been incorporated into several interventions that have successfully increased physical activity [2,3]. However, little is known about how individuals use trackers in a physical activity intervention to support behavior change.

Self-monitoring is defined as the observing and recording of one’s own behavior [4]. In the context of a behavioral intervention, the goal of self-monitoring is to increase self-awareness of target behaviors and outcomes, which has been shown to promote a range of healthy behaviors including smoking cessation [5], healthy eating and physical activity [6-8], weight management [9,10], and reducing excessive alcohol consumption [11]. There may be several benefits to self-monitoring physical activity with technology-based trackers compared with traditional self-monitoring techniques such as pedometers or self-reported recall [12,13]. Technology-based trackers automatically capture activity data, minimizing participant burden and recall bias compared with traditional paper and pencil journaling. In addition, these trackers can simultaneously monitor many indicators including steps, distance, moderate to vigorous physical activity (MVPA), heart rate, and sleep [3]. With the growing use of trackers in physical activity interventions, it is important to understand how they may help individuals self-monitor their behavior.

Trackers, and their associated mobile apps and websites, support many theory-based techniques proven to increase activity in behavioral interventions [7,8,12-17]. The behavior change techniques framework proposed by Michie and colleagues suggests that self-monitoring is the skill most strongly associated with intervention success when combined with at least one other self-regulatory technique from control theory (eg, receiving feedback on performance and reviewing progress toward goals) [7,13,15,18]. According to control theory, feedback loops provide awareness of discrepancies between performance and goals that can encourage behavior change [18]. Trackers facilitate feedback loops by providing information about physical activity behaviors in relation to individual goals. Although a key benefit of these trackers is the automatic recording of activity and personalized feedback with little burden for the wearer, it also could result in minimal engagement with the activity information. That is, simply recording activity without attending to the feedback and using additional self-regulatory techniques (ie, *passive* self-monitoring) may be insufficient to change behavior [12,15,19]. Conversely, trackers may encourage greater awareness of behavior when an individual attends to feedback provided on the tracker itself or associated mobile apps and websites (ie, *active* self-monitoring) [9,12,20-22].

Adherence to wearing a tracker may also be important for behavior change. A large body of literature suggests that strong adherence to self-monitoring of weight [23-27], diet [22,28], and physical activity [9,28] is associated with greater weight loss and improved weight control. Similarly, a systematic review found that consistent use of a pedometer is associated with higher activity levels [29]. Although some intervention studies report overall adherence or compliance to wearing a tracker (ie, proportion of total study days worn) [30-32], few have assessed patterns of adherence to tracker use *throughout* a physical activity intervention. A recent intervention trial by Cadmus-Bertram and colleagues that used a Fitbit activity tracker is one of the only published studies to take an in-depth look at adherence to wearing the Fitbit throughout the intervention [19,33]. They found adherence to wearing the Fitbit to be high and stable over time; however, it is unknown how or if participants were attending to the information collected by the trackers. There is also a lack of research examining how adherence to wearing a technology-based tracker relates to increasing physical activity. Additional research is needed to understand how best to utilize trackers in interventions to support self-monitoring and effectively change behavior.

A recently published study by Robertson et al [34] examining intervention delivery preferences found that cancer survivors are highly interested in using technology-based trackers to increase their activity. This is important as physical activity can decrease cancer recurrence [35,36], mortality [37], and improve quality of life [38,39], but unfortunately, many breast cancer survivors decrease their activity levels as much as 50% from pre- to postdiagnosis [40] and for several months to years following diagnosis [41]. Up to 65% of breast cancer survivors do not meet Centers for Disease Control and Prevention physical activity guidelines of 150 min of aerobic activity and 2 days of strength training per week [42,43]. It is important to understand how emerging, scalable intervention modalities such as technology-based trackers can be used to help cancer survivors increase their physical activity.

The goal of the current analysis was to conduct an in-depth examination of data collected from the Fitbit tracker daily for 12 weeks among a sample of breast cancer survivors enrolled in a physical activity intervention, where 7 days of ActiGraph-measured physical activity was also collected at baseline and end of study. The primary aims of this analysis were as follows: (1) examine patterns of adherence to wearing the Fitbit, (2) test the association of adherence to wearing the Fitbit with changes in ActiGraph-measured MVPA, (3) examine patterns of Fitbit-measured MVPA, (4) examine frequency of self-reported checking of data on the tracker and on the mobile app or website, and (5) test the association between self-reported checking of data on the tracker and on the mobile app or website with changes in ActiGraph-measured MVPA. We hypothesized that higher adherence to wearing the Fitbit and greater checking of the data would be associated with greater increase in ActiGraph-measured MVPA.

## Methods

### Participants and Design

Participants in this secondary data analysis were enrolled in a randomized controlled trial of a 12-week physical activity intervention. Data were collected from February 2015 to July 2016. The University of California, San Diego institutional review board approved all study procedures, and all participants provided written informed consent. The trial was registered with Clinicaltrials.gov (NCT 02332876). Eligible participants were female breast cancer survivors, in the age range of 21 to 85 years, who were diagnosed less than 5 years before study enrollment, had completed chemotherapy or radiation treatment, were sedentary (defined as self-reporting less than 60 min of MVPA in 10 min bouts per week), and had access to the Internet and a Fitbit-compatible computer, tablet, or phone. Exclusion criteria included any medical condition that could make it potentially unsafe to be in an unsupervised physical activity intervention (determined by the Physical Activity Readiness Questionnaire [44]), other primary or recurrent invasive cancer within the last 10 years, and unable to commit to a 12-week intervention.

Out of 911 women who were screened for eligibility, 108 were eligible, and 97 came to the baseline visit. Most common reasons for being ineligible included being too active (n=225), unable or unwilling to attend clinic visits (n=106), breast cancer surgery more than 5 years ago (n=81), and medical exclusion (n=36). At the baseline visit, 10 women were deemed ineligible (high blood pressure, n=8; physical limitation, n=2). A total of 87 participants were randomized to the exercise arm (n=43) or the control arm (n=44). One participant from each arm was lost to follow-up, resulting in a 97.7% retention rate (exercise n=42, control n=43) [45]. The current analyses comprise data from the 42 participants who were randomized to the exercise arm and completed the study.

A detailed description of the protocol was previously published [46]. Briefly, participants were predominantly recruited via cancer registry lists. Potential participants were telephone-screened to determine eligibility. Interested and eligible women were scheduled for an in-person visit where they were given an ActiGraph GT3X+ accelerometer to wear for 7 days and bring back to the randomization visit. At the randomization visit, participants in the exercise arm were given a Fitbit One as part of the intervention. Participants were instructed to use their Fitbit to self-monitor their physical activity. As the intervention focused on Fitbit’s “Active Minutes,” which consists of MVPA and did not focus on steps, participants were encouraged to wear the Fitbit when engaging in MVPA but were not instructed to wear it for a minimum amount of time each day. Participants were informed that their Interventionist would check their Fitbit data at least once a week and that they may be contacted by the interventionist if it seemed that they were struggling or having a great week. All participants received intervention phone calls around the 2-week and 6-week time points and automatic emails every 3 days throughout the 12-week intervention, which included reminders to sync and wear their Fitbit. One week before their final study visit, participants were mailed the ActiGraph GT3X+ and asked to wear it for 7 days, concurrently with the Fitbit, and to bring the ActiGraph to the in-person visit. At the final in-person visit, participants completed a questionnaire regarding their use of the Fitbit tracker and the Fitbit app and website.

### Measures

The Fitbit One, a commercially available accelerometer-based activity tracker, was used to examine patterns of physical activity throughout the 12-week intervention. Fitbit uses a proprietary algorithm to classify each minute as being in sedentary, light, moderate, or vigorous activity and provides metabolic equivalent of tasks (METs) for each minute. Data were wirelessly uploaded to the user’s fitbit.com account and then downloaded by the research team through a database called Fitabase (Small Steps Lab, San Diego, CA), which allows for collecting data at the minute level. Daily adherence to wearing the Fitbit tracker was defined as wearing the tracker for >10 hours in a day or logging at least some activity (>1 min MVPA). This definition for a valid Fitbit wear day was used because participants were not instructed to wear the Fitbit all day; rather they were instructed to use the Fitbit to track activity. Thus, wearing the tracker specifically to log MVPA was deemed to be valid wear based on these instructions. Fitbit wear time was determined by processing of minute level Fitbit data using the R function *accel.weartime* within the “accelerometry” package [47]. Nonwear was classified using both steps and METs. Consistent with standard protocols for ActiGraph wear time [48], greater than 90 consecutive minutes of 0 steps or METs, with 2-min tolerance (ie, for 2 min with nonzero counts during nonwear intervals) was deemed nonwear.

The ActiGraph GT3X+, a well-validated research grade accelerometer [49], provided frequency, duration, and intensity of physical activity for 7 days at baseline (prerandomization) and at week 12. Using standard guidelines, sufficient ActiGraph wear time was classified as >10 hours of wear a day for at least 5 days or >50 hours across 4 days and screened for in the ActiLife software (ActiGraph, Pensacola, FL) using guidelines outlined by Choi et al [48]. All complete and valid data were processed in ActiLife software using the low frequency extension and aggregated to 60-second epochs so that published physical activity cut points could be applied [50]. MVPA was defined as 1952 or more counts per minute (3.00-7.00 METs).

Self-report questionnaires at follow-up (12 weeks) were used to determine participants’ frequency of looking at their activity data on the Fitbit tracker itself and (in a separate question) the Fitbit app or website. These questions used an 8-point Likert scale with the following response options: more than once per day, once per day, 4-6 times per week, 2-3 times per week, once per week, 2-3 times per month, once a month or less, and never.

### Statistical Analysis

The distribution (mean [SD] and n [%]) of participant demographics and breast cancer characteristics were calculated at baseline for the analytic sample. ActiGraph-measured physical activity, measured at baseline and follow-up, was described using mean (SD). The agreement between ActiGraph- and Fitbit-measured MVPA was assessed by calculating the concordance correlation in days with overlapping wear.

#### Examine Patterns of Adherence to Wearing the Fitbit

Overall adherence to wearing the Fitbit was analyzed by determining the percent of days in the 12-week intervention period that the participant logged a valid day of wear (>10 hours wear or >1 min MVPA). Syncing errors occurred for 2 participants resulting in no data for 64 days for 1 participant and 21 days for the other participant; this data was considered missing and not classified as nonvalid.

To graphically display patterns of adherence over time, rolling adherence was calculated by determining the percent valid days in the past 6 days + the current day. To examine differences in weekly adherence, we calculated the mean (SD) of weekly adherence from the end of each week (1,...,12) and carried out a mixed effects ANOVA, with a subject level random intercept and slope and using a variance components covariance structure, to detect an omnibus difference in adherence between weeks. The subject level random intercept and slope models were used to account for the correlated nature of the weeks nested within each individual.

#### Test the Association of Adherence to Wearing the Fitbit With Changes in ActiGraph-Measured Moderate to Vigorous Physical Activity

The association between Fitbit adherence and change in ActiGraph-measured MVPA was assessed using a linear mixed effects model with a subject level random intercept. The model regressed ActiGraph-measured MVPA on overall Fitbit adherence, time (baseline vs follow-up), and the interaction between adherence and time.

#### Examine Patterns of Fitbit-Measured Moderate to Vigorous Physical Activity

Overall, Fitbit-measured MVPA was assessed by calculating the mean (SD) of day level MVPA for each participant across all valid days in the 12-week study and transforming to the week level.

To graphically display patterns of physical activity over time, rolling MVPA was calculated by summing the minutes of physical activity in the past 6 days + the current day. To examine differences in weekly MVPA, we calculated the mean (SD) of weekly MVPA from the end of each week (1,...,12) and carried out a mixed effects ANOVA, with a subject level random intercept and slope and variance components covariance structure, to detect an omnibus difference in Fitbit-measured MVPA between weeks. As before, the subject level random intercept and slope allowed us to account for the correlated nature of the weeks nested within each individual.

#### Test the Association Between Self-Reported Checking of Data on the Tracker, Mobile App, or Website With Changes in ActiGraph-Measured Moderate to Vigorous Physical Activity

Associations between physical activity self-monitoring and changes in ActiGraph-measured MVPA were analyzed using a linear mixed effects model with a subject level random intercept. The model regressed ActiGraph-measured MVPA on the self-monitoring score, time (baseline vs follow-up), and the interaction between the self-monitoring score and time. There were two “self-monitoring scores,” each based on an 8-point Likert score (one for looking at information on the Fitbit tracker and the other for looking at information on the app or website). In addition, we created a combined binary variable to assess the proportion of participants who looked at both the Fitbit tracker and the app or website daily (≥once per day) versus those who did not. The mixed effects model was run individually for each of the self-monitoring questions, with the Likert questions treated as continuous. Questions were treated as continuous because the Likert measure had 8 points, there was an underlying continuous concept (time), and low skew when the distribution was treated as continuous.

## Results

### Participant Characteristics

Participants were 42 female breast cancer survivors who were predominantly diagnosed at stage 1 (62%, 26/42). About half had received chemotherapy, and about three-fourths were currently taking an aromatase inhibitor or tamoxifen. They were an average of 58 years old (SD 11.3), with the majority being non-Hispanic (81%, 34/42), white (83%, 35/42), and having a college education or greater (69% [29/42]; Table 1). The intervention group significantly increased ActiGraph-measured MVPA from baseline (93.8 min/week, SD 90.79) to 12 weeks (195.3 min/week, SD 105.9, *P*<.001) [45]. Minutes of ActiGraph-measured MVPA at week 12 was highly correlated with Fitbit MVPA collected on overlapping days (*r*=.81: ActiGraph MVPA/day mean 29.9, SD 25.90, Fitbit MVPA/day mean 25.8, SD 28.76).

### Patterns of Adherence to Wearing the Fitbit

**Figure 1:**
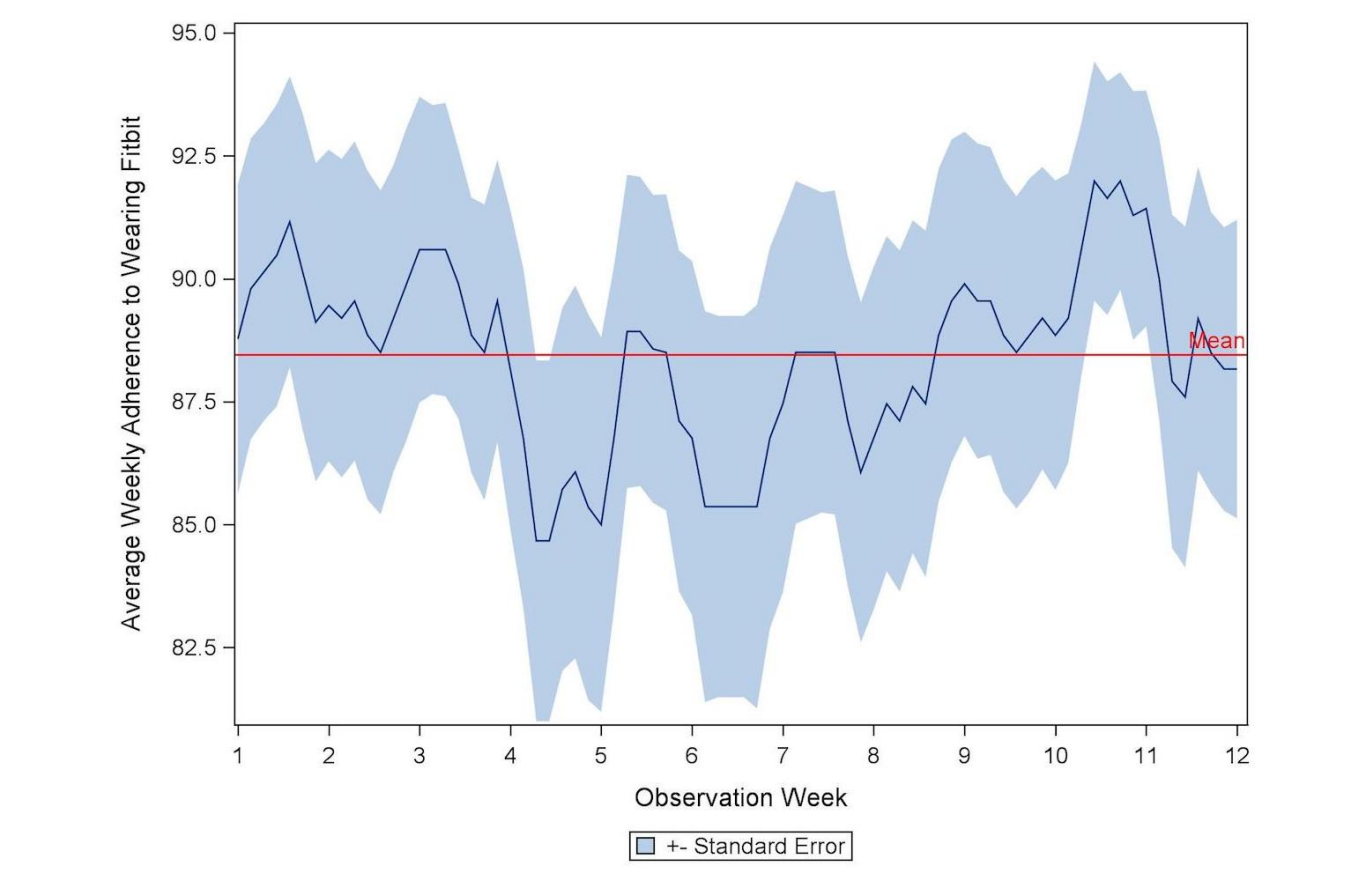
Rolling weekly percent adherence to wearing the Fitbit, averaged across study participants (standard error), reference line at overall 12 week average, n=42.

Adherence to wearing the Fitbit was high, with a mean number of valid days across the 12-week intervention period of 88% (SD 14), median of 95%, and range of 31% to 100% of intervention days. Each week, participants wore the Fitbit on average 6.2 out of 7 days (88.5% per week, SD 1.8). Although adherence to wearing the Fitbit appeared to decrease in the middle of the intervention period (Figure 1), adherence did not significantly differ across the 12 weeks (*P*=.71).

### Overall Adherence to Wearing the Fitbit and Associations With ActiGraph-Measured Moderate to Vigorous Physical Activity

Greater adherence to wearing the Fitbit was associated with greater increases in ActiGraph-measured MVPA (b_interaction_=0.35 *P*<.001). Someone with the median amount of valid wear days (95%) had an expected ActiGraph-measured MVPA increase of 109.8 min/week, whereas someone with the first or third quartile amount of valid wear days (80% and 98%, respectively) had an expected ActiGraph-measured MVPA increase of 73.3 min/week and 117.2 min/week, respectively.

### Patterns of Fitbit-Measured Moderate to Vigorous Physical Activity

**Figure 2:**
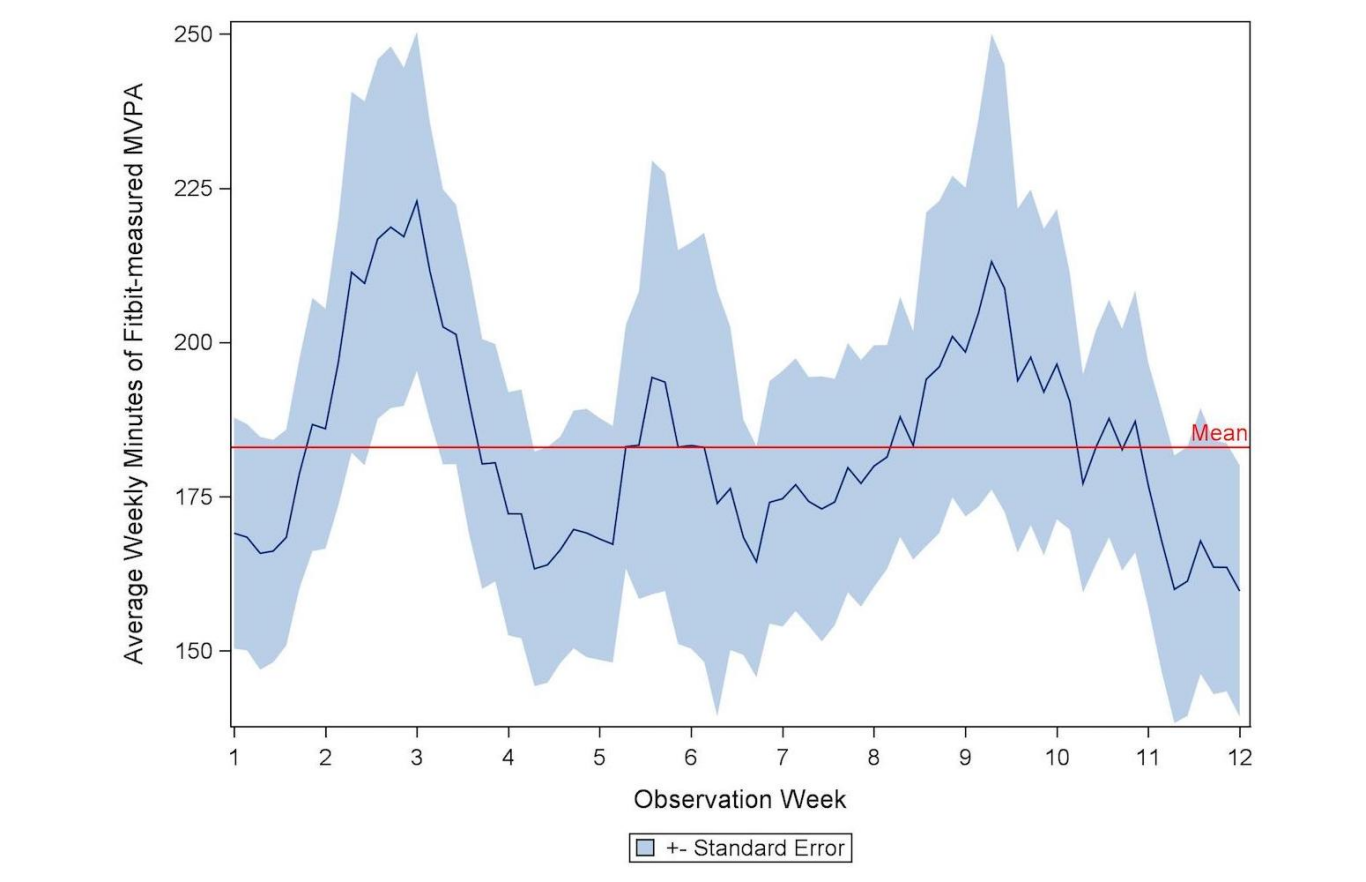
Rolling weekly minutes of Fitbit-measured MVPA, averaged across study participants (standard error), reference line at overall 12 week average, n=42.

Across the 12 weeks, participants averaged 182.6 minutes/week (SD 143.9) of MVPA on the Fitbit. Minutes of MVPA per week significantly differed over the 12 weeks (*F*_11/392_=1.91, *P*=.04; Figure 2). Weeks 3 and 9 had the highest average MVPA with 222.9 min/week (SD 173.4) and 198.4 min/week (SD 167.9), respectively. Weeks 12 and 5 had the lowest average MVPA with 159.7 min/week (SD 128.4) and 168.1 min/week (SD 123.5), respectively.

**Table 1:** Baseline characteristics (N=42).

Characteristic		Value
Age in years, mean (SD)		57.9 (11.3)
Married or living with partner, n (%)		31 (73)
Body mass index, kg/m^2^, mean (SD)		26.7 (6.3)
**Education, n (%)**		
	Some college or less	13 (31)
	College graduate	18 (43)
	Master’s degree or higher	11 (26)
**Ethnicity, n (%)**		
	Not Hispanic/Latino	34 (81)
	Hispanic/Latino	8 (19)
**Race, n (%)**		
	White	35 (83)
	Nonwhite	7 (17)
**Cancer stage, n (%)**		
	Stage I	26 (62)
	Stage II	12 (29)
	Stage III	4 (10)
Received chemotherapy, n (%)		22 (52)
Current aromatase inhibitor or tamoxifen, n (%)		30 (71)
Time since surgery, months, mean (SD)		29.5 (17.6)

### Use of the Fitbit Tracker, App, or Website

At study completion, participants answered a series of questions regarding use of the Fitbit tracker and Fitbit website or mobile app. Two participants did not answer these two items. Of the 40 participants who answered these items, 68% (27/40) reported looking at their activity data on the Fitbit app or website once a day or more; 13% (5/40) reported looking at the app or website less than once a week; 68% (27/40) of participants reported looking at the Fitbit tracker itself once a day or more, whereas 10% (4/40) reported looking at it less than once a week. Exactly half (50%, 20/40) of the participants reported looking at both the Fitbit tracker and the app or website at least once per day (Table 2).

### Association of Use of the Fitbit Tracker, App or Website With ActiGraph-Measured Moderate to Vigorous Physical Activity

Frequency of looking at one’s data on the Fitbit app or website, controlling for adherence to wearing the Fitbit, was not associated with change in ActiGraph MVPA (*P*=.36). There was a negative association between looking at one’s data on the Fitbit tracker and change in ActiGraph MVPA (b=−1.36, *P*=.07), controlling for adherence to wearing the Fitbit. Participants who reported looking at the tracker more frequently had smaller increases in MVPA than those who looked less often. When this analysis was carried out on the combined binary variable of looking at the Fitbit tracker and the app or website at least daily, we found no association with change in ActiGraph MVPA (*P*=.87).

**Table 2:** Fitbit self-monitoring questionnaires (N=40).

Question		Frequency
**Looked at information on the app or website**		
	Never	0 (0)
	Once a month or less	2 (5)
	2-3 times per month	3 (8)
	Once per week	3 (8)
	2-3 times per week	3 (8)
	4-6 times per week	2 (5)
	Once per day	6 (15)
	More than once per day	21 (53)
**Looked at information on the Fitbit tracker**		
	Never	2 (5)
	Once a month or less	1 (3)
	2-3 times per month	1 (3)
	Once per week	2 (5)
	2-3 times per week	3 (8)
	4-6 times per week	4 (10)
	Once per day	7 (18)
	More than once per day	20 (50)
**Looked at information on the app or website and on the Fitbit tracker**		
	Less than once a day	20 (50)
	Once a day or more	20 (50)

## Discussion

### Principal Findings

This study is one of the first to take an in-depth look at use of a commercially available wearable activity tracker and how it relates to changes in physical activity. Using minute level data collected from the Fitbit, adherence to wearing the tracker was high and stable across the 12-week intervention period. This is generally consistent with previous research [30,33,51]; however, one recent observational study found linear decreases in Fitbit use over a year [52]. This suggests that Fitbit use long term and not within an intervention may be different than was seen in this study. One challenge of comparing Fitbit use across studies is that how adherence to wearing the tracker was calculated or defined is often not reported [30,33,51,52]. To our knowledge, this is also one of the first studies to explore the relationship between use of a commercially available activity tracker and success in a physical activity intervention where the physical activity was also measured by an ActiGraph, the gold standard measure for free-living physical activity in research. The positive association between wearing the Fitbit and increased MVPA suggests that using the real-time data to determine if someone is wearing their tracker could help to identify individuals who may need additional support, or possibly other self-monitoring methods, to support behavior change.

In our analyses, MVPA significantly varied throughout the intervention. Interestingly, the highest minutes of MVPA occurred at week 3, immediately after the intervention call, which typically occurred around the end of week 2, and at week 9, which was around when participants were contacted to confirm their final visit at 12 weeks. This highlights the importance of personal contact with participants in a physical activity intervention and is consistent with research that has found greater benefit for combining technology-based self-monitoring with counseling than using technology alone [53].

A novel aspect of wearable trackers is that they can provide objective feedback on MVPA. Previous studies with traditional pedometers could only provide feedback on steps, which captures activities of all intensities. We identified only two other published physical activity interventions in which participants set goals explicitly on Fitbit’s active minutes and examined changes in Fitbit-measured active minutes as one of the primary study outcomes [33,54]. Given the numerous benefits of MVPA [55-57], the capability of trackers to automatically collect information on MVPA may be useful in helping individuals meet physical activity guidelines [13].

While technology-based activity trackers make self-monitoring less burdensome compared with traditional tracking methods, they also do not require a person to attend to the information being collected by the monitor. Overall, self-reported viewing of activity data on the Fitbit itself, or on the Fitbit website or app was very high. Looking at activity data on the app or website was not associated with changes in ActiGraph-measured MVPA. Surprisingly, more frequent looking at data on the Fitbit tracker itself was associated with smaller changes in ActiGraph-measured MVPA. One reason for this finding may be that the Fitbit One tracker did not show minutes of MVPA; that information was only available on the app or website. These results could also indicate that checking one’s own data is not as important as being accountable to someone else for increasing physical activity. In this study, it was stressed that the Fitbit would be used so that the interventionist could see the data and provide support. It may be that being accountable was a greater motivating factor for increasing MVPA than being self-aware of one’s own activity levels. Much of the field’s understanding of the importance of self-monitoring is based on active self-monitoring, which typically require a person to think about their day and record their minutes of activity, but this record is often not easily or immediately shared. As technology-based trackers become more common place in interventions, we need to continue exploring the impact of active versus passive self-monitoring and the role of accountability on behavior change so that our understanding of the role of monitoring physical activity is consistent with new and emerging technologies.

### Limitations

Although this in-depth analysis of daily activity data from a commercially available activity tracker is an important addition to our understanding of how trackers are associated with behavior change, several limitations should be noted. This study comprised a small sample of a relatively homogenous group of breast cancer survivors, and results may not be generalizable. Fitbit does not share information on number of times a person checks the Fitbit app or website; therefore, checking of app or website relied on self-report, which unfortunately had little variation, with most participants reporting looking at their Fitbit or the app or website daily. Additionally, self-report questions assessing use and engagement with the tracker were only asked at the end of the intervention, limiting our ability to examine trends in engagement throughout the intervention. In addition, we used a question combining website and app use and could not examine those two modalities separately. The intervention period was relatively short—long-term use of an activity tracker and its relationship with increasing physical activity could not be assessed. Although we also used standard cut-points for determining ActiGraph-measured MVPA, future studies should consider using machine learning algorithms to classify ActiGraph-measured behaviors. Finally, the intervention included many reminders to wear the Fitbit, so adherence results may not be representative of what would happen outside of an intervention protocol.

### Conclusions

With the continued emergence of new technologies for self-monitoring physical activity, it is important to understand how people use these new devices and how use of these devices can support behavior change. These insights may enhance our ability to effectively utilize activity trackers to promote behavior change.

## Abbreviations

MET: metabolic equivalent of task
MVPA: moderate to vigorous physical activity

## Appendix

Multimedia Appendix 1 CONSORT E-HEALTH checklist (V 1.6.1).
